# First Report of *CTNS* Mutations in a Chinese Family with Infantile Cystinosis

**DOI:** 10.1155/2015/309410

**Published:** 2015-03-17

**Authors:** Yong-jia Yang, Yuan Hu, Rui Zhao, Xinyu He, Liu Zhao, Ming Tu, Lijun Zhou, Jihong Guo, Linqian Wu, Tantai Zhao, Yi-min Zhu

**Affiliations:** ^1^The Laboratory of Genetics and Metabolism, Hunan Children's Research Institute (HCRI), Hunan Children's Hospital, University of South China, Changsha 410007, China; ^2^The Special Inspection Department, Hunan Children's Research Institute (HCRI), Hunan Children's Hospital, University of South China, Changsha 410007, China; ^3^State Key Laboratory of Medical Genetics, Central South University, Changsha 410008, China; ^4^Department of Ophthalmology, Xiangya 2nd Hospital, Central South University, Changsha 410013, China

## Abstract

Infantile cystinosis (IC) is a rare autosomal recessive disorder characterized by a defect in the lysosomal-membrane transport protein, cystinosin. It serves as a prototype for lysosomal transport disorders. To date, several *CTNS* mutations have been identified as the cause of the prototypic disease across different ethnic populations worldwide. However, in Asia, the *CTNS* mutation is very rarely reported. For the Chinese population, no literature on *CTNS* mutation screening for IC is available to date. In this paper, by using the whole exome sequencing and Sanger sequencing, we identified two novel *CTNS* splicing deletions in a Chinese IC family, one at the donor site of exon 6 of *CTNS* (IVS6+1, del G) and the other at the acceptor site of exon 8 (IVS8-1, del GT). These data give information for the genetic counseling of the IC that occurred in Chinese population.

## 1. Introduction

Cystinosis is a rare autosomal recessive disorder characterized by the intralysosomal accumulation of the disulphide amino acid cystine, which is the consequence of a defect in the membrane transport protein, cystinosin [1, 2].

The cystinosin was coded by* CTNS* gene, which consists of 12 exons, is located on chromosome 17p13.3, and spans 23 kb of genomic DNA [1, 3].

The cystinosis serves as the prototype of inborn error for a small group of lysosomal transport disorders as it was the first described lysosomal storage disease by distinguished European pediatrician Guido Fanconi in early 1930 [3].

Three subtypes of cystinosis have been described according to the age of onset and the severity of the clinical symptoms: infantile cystinosis (IC, the classical and the severest form, OMIM #219800), juvenile cystinosis (the intermediate form, OMIM #219900), and ocular nonnephropathic cystinosis (OMIM #219750) [3].

Since the* CTNS* gene was cloned as the cause of cystinosis [1], a great number of* CTNS* mutations spreading throughout the entire gene, including small insertion, deletion, duplication, point mutation, splice-site mutations, promoter mutations, and genomic rearrangements, have been reported, mostly in European- and American-based subpopulations [3–5].

Interestingly, in Asia population, limited cases with cystinosis were diagnosed and limited* CTNS* mutations were reported [6–9].

Specifically, in the most populated Chinese population, there was only one Taiwan family with two sisters affected by intermediate cystinosis reported, which was the consequence of a homozygous missense mutation (N323K) of* CTNS* gene [7, 10].

In here, we described, for the first time, a Chinese mainland family with two brothers affected by IC diagnosed by exome sequencing. All of the two novel* CTNS* mutations were involved in the deletions of the splice-sites of CTNS (IVS6+1, del G and IVS8-1, and del GT).

## 2. Materials and Methods

**Figure 1:**
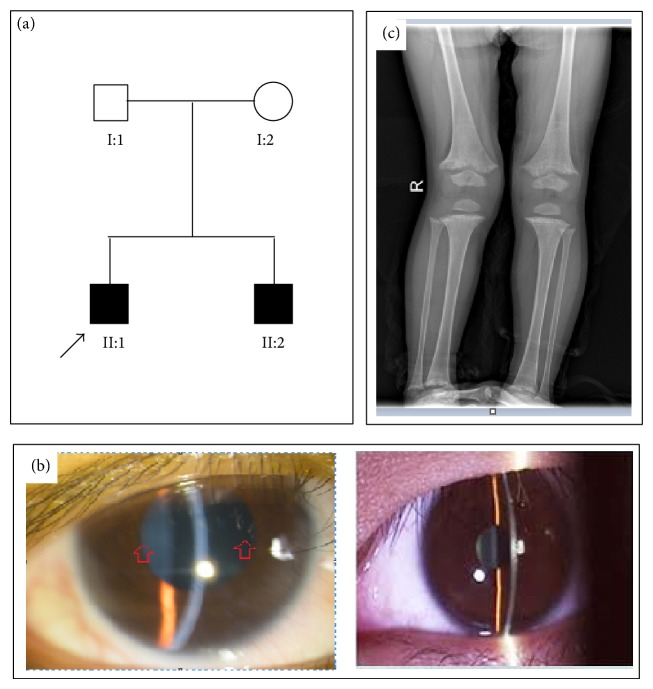
The characteristic clinical features of the family with infantile cystinosis. (a) The family investigated in this study. (b) The Corneal crystals on Slit Lamp Examination (left: the proband; right: the control). (c) The radiological signs of renal rickets in the proband.

A Chinese Han family (from Hunan Province, Central China) with two males affected by renal Fanconi syndrome (Figure 1(a)) and 80 unrelated ethnically matched healthy controls (41 male and 39 female) were recruited in this study.

All adult individuals and the parents of the minors who participated in this study gave written informed consent, which was approved by the Ethics Committee of the Hunan Children's Hospital, Changsha City, China. The procedures of the Committee conformed to the principles of the declaration of Helsinki, 2008 edition.

For exome capture, genomic DNA was extracted from peripheral blood (4 mL in heparin sodium tubes) using the phenol/trichloromethane method prescribed by standard protocol. A total of 3 micrograms (ug) of genomic DNA (one patient, II:1, Figure 1(a)) was sheared by sonication and hybridized to the Nimblegen SeqCap EZ Library for enrichment, according to the manufacturer's protocol. The library enriched for target regions was sequenced on the HiSeq 2000 platform to get paired-end reads with read length of 90 bp [11]. A mean exome coverage of 73.66x was obtained that provided sufficient depth to accurately call variants at 99% of each targeted exome. For read mapping and variant analysis, the human reference (genome version hg19, build 37.1) was obtained from the UCSC database (http://genome.ucsc.edu/). Sequence alignment was performed using the program SOAP aligner. SNPs were called using SOAPsnp set with the default parameters after the duplicated reads (produced mainly in the PCR step) were ignored [12]. Short insertions or deletions (indels) affecting coding sequence or splicing sites were identified. The thresholds for calling SNPs and short indels included the facts that the number of unique mapped reads supporting a SNP had to be ≥C4 and the consensus quality score had to be ≥C20 (the quality score is a Phred score, generated by the program SOAPsnp1.03; quality score 20 represents 99% accuracy of a base call). Common mutations of the patient were obtained and were filtered against the dbSNP137, 1000 Genomes project, HapMap project, Exome Sequencing Project (ESP) and our inhouse databases with a frequency >0.005.

Due to the absence of consanguineous marriage identified in the family and two family members exhibiting a similar phenotype of renal Fanconi syndrome, we firstly focused on the candidate-genes of compound-heterozygous mutations. Sanger sequencing was employed to validate the identified potential disease-causing variants with ABI3500 Genetic Analyzer (Applied Biosystems, Foster City, CA, USA) [13]. The primers and PCR conditions were available on request.

## 3. Results

### 3.1. Clinical Data

The propositus (II:1, Figure 1(a)) was the first child of nonconsanguineous parents. He was 3050 g in weight and 47 cm in length at birth and had undergone a full-term pregnancy and normal vaginal delivery. The pregnancy was uncomplicated (no exposures to or use of alcohol, tobacco, and drugs), but the gravida was affected by intrahepatic cholestasis.

The Initial symptom of the propositus was persistent vomiting at the age of 7 months. At that time, his examinations of gastrointestinal B-ultrasound, upper gastrointestinal barium meal, gastrointestinal endoscope, brain MRI, and EEG were at the normal range. His result for chromosome G band analysis (550 bands level) was 46, XY.

At the age of 1 year and 7 months, his symptom of polyuria appeared (he need to intake 2000–2500 mL water daily). His hemoglobin was 87 g/L (normal: 110–180 g/L); his blood amino acid analysis showed nutritional disorders, secondary carnitine deficiency, and ketosis. A subsequent initiation of lactose-free diet, supplemented with carnitine, VitB1, and VitB12, alleviated his vomiting, but his polydipsia and polyuria remained unchanged. At the age of 3 years and 6 months, he was diagnosed to be affected by renal Fanconi syndrome, metabolic acidosis (severe), anemia (moderate), renal rickets, hypokalemia, and generalized amino aciduria.

At the age of 7 years and 6 months, he was diagnosed to be affected by IC by exome sequencing. His height was 100.5 cm (<5 centile, normal: 120.7 cm); he showed severe Corneal crystals (Figure 1(b)) and severe renal rickets (Figure 1(c)).

The patient II:2 (Figure 1(a)) was the younger brother of the propositus II:1 and was 3 years and 4 months old. His initial symptom was glycosuria at the age of 7 months. The urinalysis revealed: glucose: +++, urine protein: ++. The blood chemistry results were pH 6.0 (normal: 7.35–7.45), potassium 2.7 mmol/L (normal: 3.5–5.5 mmol/L), and bicarbonate 10.74 mmol/L (normal: 20–29 mmol/L). From age of 1 year and 5 months, he exhibited polydipsia and polyuria (water intake 3000 mL; urinates 3300 mL daily). He begins to suffer from constipation and his appetite is very poor. Anthropometric evaluation revealed a short stature (height of 81 cm, normal: 92.5 cm) but intelligence is at normal range. He was diagnosed to be affected by renal Fanconi syndrome, metabolic acidosis, hypokalemia, iron deficiency anemia, secondary carnitine deficiency, and Vit D-dependent rickets at the age of 9 months.

### 3.2. Exome Sequencing and Sanger-Sequencing

We performed exome sequencing of one patient (II:1, Figure 1(a)) in the Chinese family with the symptoms of renal Fanconi syndrome. We generated 6.5 billion bases of 90 bp paired-end read sequence for the patient. About 6.2 billion (96.7%) of the bases passed the quality assessment, 5.5 billion (89.2%) aligned to the human reference sequence, and 3.0 billion bases (54.2%) mapped to the targeted bases with a mean coverage of 64.1-fold. We excluded known variants identified in 1,000 genome project, HapMap, dbSNP132, or YH1 [11].

In the family, due to the nonconsanguineous marriage and two brothers being affected by the same severe syndrome, we mainly focused on the candidate-gene that must meet the following three criteria: (1) the candidate must contain at least two nonhomozygous variants (for the criteria, we obtained only 17 candidate genes, Table 1); (2) the candidate genes must contain one truncated or splice-site mutated allele (for the criteria, we obtained only 2 candidate genes,* CTNS* and* MYH15*, Table 1); (3) the candidate variants must be confirmed by Sanger sequencing and must be cosegregated with the renal Fanconi symptoms in the family; for this criteria, we discovered that only two splice-site deletions of* CTNS* were cosegregated with the syndrome in the family with the autosomal recessive mode (Figure 1(a)), while the MYH15 variants shared no cosegregation in the family as none of the MYH15-variants was detected in the patient II:2.

Further Sanger sequencing of CTNS gene was realized on 80 ethnic-matched, healthy controls and revealed that none of controls carried the CTNS mutation.

**Figure 2:**
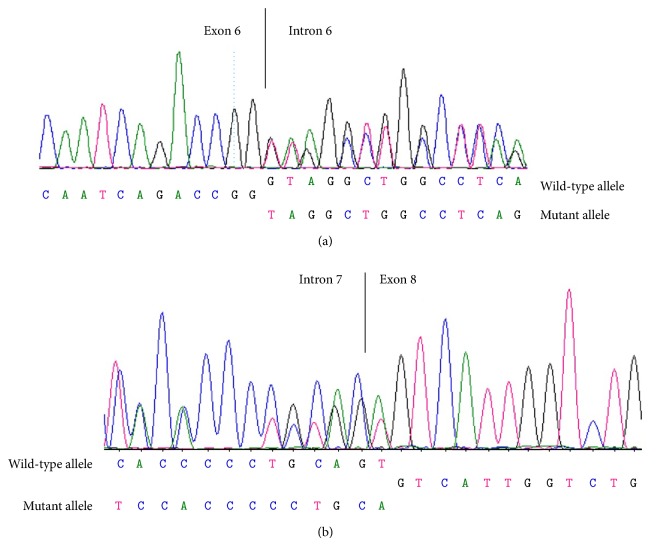
Two splice-site deletions of CTNS identified in this study. (a) IVS6+1, del G. In genome: chr17, del3558396G. (b) IVS8-1, del GT. In genome: chr17 del3559780-81Gt (note: this mutation strikes twice, firstly, it leads to IVS8-1, del G; secondly, it leads to the c.462delT).

All two* CTNS* mutations detected in this family were involved in splice-site deletion, one at the donor site of exon 6 of* CTNS* (IVS6+1, del G) (Figure 2(a)) and the other at the acceptor site of exon 8 (IVS8-1, del GT) (this mutation strikes twice; firstly, it leads to IVS8-1, del G; secondly, it leads to the c.462delT) (Figure 2(b)).

## 4. Discussion

The majority of diagnosed cystinosis cases were reported in the European and American population with an incidence of one in 100,000–200,000 live births [3].

In France's Brittany province, especially, the estimated incidence of cystinosis was 1 case per 25,909 live births [3, 4, 14].

About 76% of cystinosis patients in European and American population carried the common 57 kb multiexon deletion, while in France's Brittany, with special high incidence with cystinosis, the founder effects of 898–900+24 del27 and W183X mutation exactly existed [14].

Interestingly, in Asia population, very few cystinosis patients and* CTNS* mutations have been reported. To date, only one case in Japan [8], six cases (four families) in Thailand [6], two cases in India [15] (Tang et al., 2009), one family (two affected sisters with intermediate cystinosis) in Taiwan (which represents Chinese population) [7], and several cases in Iran [9] have been reported.

Since the availability of cystine-depleting medical therapy and the introduction of kidney transplants, the previously fatal disease was recently transformed into a treatable disorder [9, 16].

A recent study has pointed out that a huge gap of the outcomes for the nephropathic cystinosis patients existed between developed and developing countries as the limited access to cysteamine [17]. The authors noticed that the main obstacle on the way for the IC-therapy in China was that the IC patients were not diagnosed which as the consequence of the unavailable of genetic and clinical data for this population.

China was the most populated developing country in the world, to our knowledge; except for one family (two affected sisters with a* CTNS* homozygous mutation N323K) in Taiwan (which originated from Chinese population) with intermediate cystinosis reported [7], no IC patient was reported in this developing country.

In this study, due to the limited genetics information for* CTNS* genotypic phenotype available in Chinese population, we firstly carried out the mutation screening of* SLC34A*3 [18] and SLC34A1 [19] (which independently cause recessive renal Fanconi syndrome) in the family but negative results were obtained (data not shown). Although the negative data observed, we carried out the Whole Exome Sequencing in the family. By exome sequencing, we successfully diagnosed the infantile cystinosis in the family and identified two novel* CTNS* splice-site deletions in the family. This is the first report where IC in Chinese population and the first time of* CTNS* splice-site deletion were detected. These data provide information for the molecular diagnosis of cystinosis in the Chinese population and give clues for the* CTNS* mutation distribution and classification in the world.

## Figures and Tables

**Table 1:** The candidate-gene list of all the genes with compound heterozygous mutations, which were detected by Exome sequencing.

Chromosome	Position	Reference	Gene name	Detailed information for case m0957	MutType
chr16	2376053	C	ABCA3	Y99T13C6,0000,missense	SNP
chr16	2376215	G	ABCA3	S99C21G16,0000,missense	SNP
chr7	2969678	G	CARD11	K99G13T12,0000,missense	SNP
chr7	2969679	C	CARD11	Y99C12T11,0000,missense	SNP
chr17	3558396	G	CTNS	−1G;Het;7/17;—,—,—,—;splice-site	Indel
chr17	3559780	GT	CTNS	−2GT;Het;17/37;—,—,—,—;splice-site	Indel
chr6	65596690	T	EYS	K99G38T36,0000,missense	SNP
chr6	65596696	G	EYS	S99G37C37,0000,missense	SNP
chr1	89597644	C	GBP7	M21C4A2,0000,3-UTR	SNP
chr1	89614981	C	GBP7	M99A34C29,0000,missense	SNP
chr2	121555032	G	GLI2	R99G17A14,0000,missense	SNP
chr2	121745815	G	GLI2	R20G5A2,0000,3-UTR	SNP
chr5	63256212	G	HTR1A	R99G15A14,0000,3-UTR	SNP
chr5	63256702	C	HTR1A	Y99T11C6,0000,missense	SNP
chr4	6577044	A	MAN2B2	R57A3G3,0000,missense	SNP
chr4	6621751	C	MAN2B2	Y99T20C19,0000,missense	SNP
chr7	100642828	C	MUC12	Y62C10T4,0000,missense	SNP
chr7	100647142	G	MUC12	R99G73A49,0000,missense	SNP
chr3	108220603	C	MYH15	Y99C13T13,0000,missense	SNP
chr3	108224685	—	MYH15	+1A;Het;10/22;−1,—,—,—;splice-site	Indel
chr11	48510472	T	OR4A47	Y99T28C13,0000,missense	SNP
chr11	48510952	T	OR4A47	K99G18T12,0000,missense	SNP
chr5	140700	G	PLEKHG4B	R99A6G6,0000,missense	SNP
chr5	169624	G	PLEKHG4B	R99G19A16,0000,missense	SNP
chr8	27295302	G	PTK2B	S99C19G18,0000,NR_exon	SNP
chr8	27300416	C	PTK2B	M99C16A15,0000,missense	SNP
chr6	167592605	T	TCP10L2	K99T39G8,0000,missense	SNP
chr6	167592606	T	TCP10L2	W99T39A8,0000,missense	SNP
chr19	54665989	C	TMC4	Y99C11T11,0000,missense	SNP
chr19	54666990	G	TMC4	R48G8A3,0000,NR_exon	SNP
chr8	30612299	C	UBXN8	Y99C31T18,0000,missense	SNP
chr8	30620846	A	UBXN8	R65A9G4,0000,readthrough	SNP
chr9	104162272	A	ZNF189	R99G19A19,0000,missense	SNP
chr9	104170748	A	ZNF189	R99A20G21,0000,missense	SNP
